# The Combination of Vibrational Spectroscopy and Chemometrics for Analysis of Milk Products Adulteration

**DOI:** 10.1155/2021/8853358

**Published:** 2021-06-29

**Authors:** Anjar Windarsih, Abdul Rohman, Irnawati, Sugeng Riyanto

**Affiliations:** ^1^Research Division for Natural Product Technology (BPTBA), Indonesian Institute of Sciences (LIPI), Yogyakarta 55861, Indonesia; ^2^Center of Excellence, Institute for Halal Industry and Systems (PUI-P IHIS), Universitas Gadjah Mada, Yogyakarta 55281, Indonesia; ^3^Department of Pharmaceutical Chemistry, Faculty of Pharmacy, Universitas Gadjah Mada, Yogyakarta 55281, Indonesia; ^4^Faculty of Pharmacy, Universitas Gadjah Mada, Yogyakarta 55281, Indonesia; ^5^Faculty of Pharmacy, Halu Oleo University, Kendari 93232, Indonesia

## Abstract

Milk products obtained from cow, goat, buffalo, sheep, and camel as well as fermented forms such as cheese, yogurt, kefir, and butter are in a category of the most nutritious foods due to their high contents of high protein contributing to total daily energy intake. For certain reasons, high price milk products may be adulterated with low-quality ones or with foreign substances such as melamine and formalin which are added into them; therefore, a comprehensive review on analytical methods capable of detecting milk adulteration is needed. The objective of this narrative review is to highlight the use of vibrational spectroscopies (near infrared, mid infrared, and Raman) combined with multivariate analysis for authentication of milk products. Articles, conference reports, and abstracts from several databases including Scopus, PubMed, Web of Science, and Google Scholar were used in this review. By selecting the correct conditions (spectral treatment, normal versus derivative spectra at wavenumbers region, and chemometrics techniques), vibrational spectroscopy is a rapid and powerful analytical technique for detection of milk adulteration. This review can give comprehensive information for selecting vibrational spectroscopic methods combined with chemometrics techniques for screening the adulteration practice of milk products.

## 1. Introduction

In the last decades, the production and consumption of dairy products have increased globally. The market growth of the dairy market is expected to continuously rise up to 2025, with annual production growth of 2.0% for skim milk powder, 2.1% for whole milk powder, 1.7% for butter, and 1.4% for cheese. Due to high nutritional values, the dairy products in the form of either raw milk or lacteous derivatives are highly susceptible to adulteration practices and frauds [1]. The adulteration of dairy products can be done by replacing a component with a similar but cheaper alternative, introducing illegal substances such as addition of melamine, spuriously extending shelf-lives, declaring incorrect or no processes during food production, making incorrect claims about the types of ingredients used and quantitative information of food components, and providing incorrect information about geographical source [2]. The adulteration of food products which involves the addition or substitution of low-quality ingredients into high-quality ones or the removal of crucial ingredients of food may be probably as old as food manufacturing. Indeed, consumers demand the accurate and correct food labelling to select food products [3].

The selection of food by consumers is typically dictated either by lifestyle (halal foods free from forbidden components such as pork, vegetarians preferring organic foods) or by health concerns especially due to allergenic reactions such as consumer's preference for food products without ingredients like lactose and gluten. Several adulteration issues on dairy products have been reported in recent years. The presence of adulterants or foreign substances which are different from those declared in labelled products is a serious matter for all stakeholders including consumers, producers, and regulatory agencies [4]. Therefore, food authentication analysis is needed to assure the quality of products.

Food authentication is the process by which the food products are regularly checked for quality, safety, and the correctness with their description on labelled products using standardized methods. Authentication of milk products involves some analytical methods capable of confirming that the milk product matches the stated labels which conform some laws and regulations [5].

Numerous chemical, physical, or biological analytical methods, including the analysis of ingredients and determination of geographical origin intended for authentication analysis in dairy products, are reported by food scientist [6]. Among these methods, vibrational spectroscopies are one of the most reported ones. Due to the complex spectra obtained during the analysis of samples, the chemometrics analysis is typically used to assist the vibrational spectra to make them more easily interpretable. This review highlights the recent updates on the application of vibrational spectroscopy combined with chemometrics techniques for authentication of dairy products based on articles published during 2010-2020.

## 2. Chemometrics

The term “chemometrics” was coined several decades ago by Bruce Kowalski to describe a new way of analyzing chemical data, in which elements of both statistical and chemical thinking are combined [7]. Many definitions of chemometrics are available. According to one of these definitions, the chemometrics is “a scientific discipline where chemistry and pharmaceutical science meet statistics and software” [8]. According to the International Chemometrics Society, chemometrics is “(i) the chemical discipline that uses mathematical and statistical methods to design or select optimal measurement procedures and experiments (ii) to provide maximum chemical information by analyzing chemical data” [9]. The commonly used methods of chemometrics or multivariate analysis are hierarchical and nonhierarchical cluster analysis, principal component analysis (PCA) for classification, multilinear regression (MLR), partial least square (PLS), and principal component regression (PCR) for multivariate regression [9, 10].

### 2.1. Hierarchical Cluster Analysis (HCA)

The hierarchical cluster analysis is a method to evaluate the distance between samples and groups in a plot namely dendrogram. In this method, the different equations can be used to calculate the distance, such as Euclidean (equation (1)) or Mahalanobis (equation (2)) or Manhattan (equation (3)) distance [9].

(1) $$\begin{array}{c} \text{Distance}=\sqrt{{\left(X_{1}-Y_{1}\right)}^{2}+{\left(X_{2}-Y_{2}\right)}^{2}+\cdots +{\left(X_{n}+Y_{n}\right)}^{2}}, \end{array}$$

where *X*_*n*_ and *Y*_*n*_ are the coordinates of sample *X* and *Y* in the *n*^th^ dimension of row space.

(2) $$\begin{array}{c} \text{Distance}=\sqrt{{\left(X_{i}-Y_{j}\right)}^{T}C^{-1}\left(X_{i}+Y_{j}\right)}, \end{array}$$

where *X*_*i*_ and *Y*_*j*_ are column vectors for objects *i* and *j*, respectively, and *C* is the covariance matrix.

(3) $$\begin{array}{c} \text{Distance}=\overset{p}{\underset{i=1}{\sum }}\left|X_{i}-Y_{i}\right|, \end{array}$$

where *X*_*i*_ and *Y*_*i*_ are vectors.

### 2.2. Principal Component Analysis (PCA)

The principal component analysis is a feature for reducing the amount of data when there is a correlation present among group of samples. PCA is a useful technique if the variables are correlated. The idea behind PCA is to find principal components *Z*_1_, *Z*_2_, ⋯, *Z*_*n*_, which are linear combinations of the original variables describing each specimen, *X*_1_, *X*_2_, ⋯, *Xn*, i.e., latent variables.

(4) $$\begin{array}{c} Z_{1}=a_{11}X_{1}+a_{12}X_{2}+a_{13}X_{3}+\cdots a_{1n}X_{n}, \end{array}$$

(5) $$\begin{array}{c} Z_{2}=a_{21}X_{1}+a_{22}X_{2}+a_{23}X_{3}+\cdots a_{2n}X_{n}. \end{array}$$

The new variables were chosen following the coefficients, *a*_11_, *a*_12_, etc., unlike the original variables. The PCA is also the decomposition of a data set into principal components (PCs). The first principal component (PC1), *Z*_1_, accounts for most of the variation in the data set, and the second principal component (PC2), *Z*_2_, accounts for the next most considerable variation. The number of useful PCs is much less than the number of original variables if significant correlation occurs [10].

### 2.3. Multiple Linear Regression (MLR)

MLR is used to establish linear relationships between multiple independent variables and the dependent variables. MLR can be described as a regression equation for any *n* components as shown in equation (6).

(6) $$\begin{array}{c} A=\varepsilon_{1}{bc}_{1}+\varepsilon_{2}{bc}_{2}+\varepsilon_{1}{bc}_{1}+\varepsilon_{3}{bc}_{3}+\cdots +\varepsilon_{n}{bc}_{n}. \end{array}$$

The equation produced by MLR can be used for quantitation [11].

### 2.4. Principal Component Regression (PCR)

PCR is a combination of PCA and MLR. The basic PCR is to reduce the number of predictor variables by using their first few principal components rather than the original variables. The method works well when there is a considerable degree of correlation between the predictor variables. PCR is also a useful method when the predictor variables are very highly correlated [10].

### 2.5. Partial Least Square (PLS)

PLS regression uses linear combinations of the predictor variables rather than the original variables. However, the way in which these linear combinations are chosen is different. In PLS, variables that show a high correlation with the response variables are given extra weight because they will be more effective at prediction. In this way, linear combinations of the predictor variables are chosen that are highly correlated with the response variables and also explain the variation in the predictor variables [10].

The application of chemometrics or multivariate data analysis (MDA) is emerging in the authentication of milk using FTIR spectroscopy (Irnawati et al., 2021). Several studies have reported using chemometrics for classification of milk such as the authentication of raw milk from reconstituted milk with FTIR spectroscopy combined partial least square-discriminant analysis (PLS-DA) [12], the detection of formalin in cow milk using ATR-FTIR spectroscopy combined with PCA and its quantification using PLS [13], and the detection and quantification of urea in milk using FTIR spectroscopy combined with multivariate analysis (PCA and PLS) [14]. Capuano et al. [15] have reported using FTIR spectra and PLS-DA for classification of milk from cow that were fed with or without fresh grass. Windarsih et al. [16] have reported using FTIR spectroscopy combined with chemometrics (PLS) for authentication of Bovine Milk Fat (BMF) from Lard Oil (LO).

Bergamaschi et al. [17] have compared the use of infrared spectra, fatty acid profiles, flavor fingerprints, and sensory descriptions for authentication of farming systems to determine the origin of milk. The results showed that the FTIR spectra combined with linear discriminant analysis have been proven to be valuable instruments for obtaining information on the farming system in which the milk is produced. Liu et al. [18] have also reported the use of near-infrared spectroscopy combined with linear discriminant analysis for the authentication of organic milk.

### 2.6. Application of Vibrational Spectroscopy and Chemometrics for Authentication of Milk

According to FFDCA (Federal Food, Drug and Cosmetic Act) of Food and Drug Administration (FDA), milk can be declared as “adulterated” due to (a) the addition of substances which are harmful to human health such as melamine, (b) the addition of cheaper or inferior quality milk into high priced quality milk, (c) the extraction of any valuable components from milk, (d) the reduced quality of milk which is below the required standards, and (e) the addition of any substances in order to increase bulk or weight such as sucrose and maltodextrin [19].  Table 1 lists the applications of vibrational spectroscopy (Raman, near infrared, and mid infrared) combined with chemometrics for milk authentication.

### 2.7. Addition of Milk from Different Sources

Several vibrational spectroscopic techniques have been reported for the analysis of milk adulteration from various sources. Near-infrared (NIR) spectroscopy combined with PLS algorithms was used for the analysis of goat milk (GM) adulteration with cow milk (CM). The addition of CM into GM can be a serious matter because CM can represent a health problem, especially for allergic consumers regardless of its consumed amount. PLS-DA using variables from absorbance values belonging to 10000-4000 cm^−1^ range was able to discriminate GM and GM added with CM in amounts as low as 1.0154 g/100 g, with accuracy of discrimination of 100%. For the quantification of adulterants (CM), the multivariate calibration of Successive Projections Algorithm for interval selection in PLS (iSPA-PLS) provided the best results exploiting 13 point-moving mean and baseline offset with coefficient of correlation (*r* value) of 0.9996 for the correlation between actual values and predicted values and RMSECV of 0.8016 g/100 g and RMSEP value of 3.6597 g/100 g with relative error of prediction of 11.24%. This indicated that by selecting appropriate spectral treatment, NIR spectra combined with iSPA-PLS offered rapid and accurate technique for the authentication of GM from CM [20].

Analysis of CM in GM was also performed using Fourier transform infrared (FTIR) and Raman spectroscopy combined with chemometrics methods, SIMCA, and partial least square (PLS) regression. For FTIR measurement, the spectra were recorded from wavenumber of 4000-650 cm^−1^ using 64 interferogram and resolution of 4 cm^−1^. SIMCA as a chemometrics method could completely separate between authentic and adulterated GM with CM, except in 5% of CM concentration. Samples containing high concentration of CM appeared close to the pure CM and categorized as adulterated samples. PLS regression was used for predicting the concentration of CM in GM precisely and accurately, and the model had a high value of *R*^2^ calibration (0.97) and *R*^2^ validation (0.98) and also a low value of SECV (7.5) and SEP (5.9). On the other hand, for Raman measurement, samples were measured using Raman spectroscopy equipped with a laser source of 1064 nm. Spectra acquisition was carried out at the wavenumber of 1850-250 cm^−1^ using a resolution of 4 cm^−1^ and integration time of 2500 ms. The combination of Raman spectroscopy with SIMCA also demonstrated good classification between pure GM and adulterated GM with CM. Moreover, PLS regression was successfully employed for predicting concentration of CM in GM with *R*^2^ calibration of 0.97 and *R*^2^ validation of 0.98 with lower SECV and SEP values accounting for 7.3 and 6.9, respectively [21].

Camel milk contains some important nutritional components especially protein. Due to its high price compared to other milks, it is very potential to be adulterated with other lower price milks. For instance, the price of camel milk is three times higher than that of cow milk; therefore, it is often adulterated with cow milk by producers for economic reasons. The authentic and adulterated samples of camel milk were subjected to FTIR spectrophotometer measurement using attenuated total reflectance (ATR) technique and recorded as absorbance. The acquisition of spectra was performed in the wavenumber of 4000-650 cm^−1^ with the number of scans of 98 and resolution of 4 cm^−1^. Prior to chemometrics analysis, the spectra went through pretreatment techniques, namely, scatter correction (standard normal variate and normalization), and Savitzky-Golay derivatization to obtain a good chemometrics model. PLS calibration in the wavenumber of 3000-920 cm^−1^ was successfully used for the detection and quantification of cow milk in camel milk which obtained *R*^2^, and RMSEC in the calibration model was 0.9939 and 0.9322, respectively. The validation model demonstrated high a *R*^2^ value (0.9922) and low value (1.0618) of RMSEP which confirmed the validity of the PLS model to be used for cow milk quantification in camel milk. The model showed low relative error (3.8%) and low LOD value of 2.595% and was proposed to be an adequate method for the authentication of camel milk from cow milk [22].

Adulteration of raw milk from reconstituted milk has been investigated using FTIR spectroscopy in combination with PLS-DA. Milk samples were lyophilized using a freeze drier to remove the water content. Samples were then measured using FTIR spectroscopy in the wavenumber of 4000-650 cm^−1^ with a resolution of 60 and an interval of 0.48 cm^−1^. The PLS-DA model was created using first derivative spectra in the wavenumber region of 1800-800 cm^−1^. Data used for variables were subjected to autoscaling prior to building the PLS-DA model. Result demonstrated that the PLS-DA model successfully classified raw milk and raw milk adulterated with reconstituted milk. The lowest adulterant concentration which still could be predicted by the PLS-DA model was 10%. The PLS-DA model was validated using both internal and external validations. The aim of validation is to identify whether the PLS-DA model is overfitting or not. Model overfitting is avoided because it affects the classification performance and gives bias results. An internal and external validation test using 11 latent variables confirmed the validity of the PLS-DA model with the accuracy of 98% [12].

**Figure 1:**
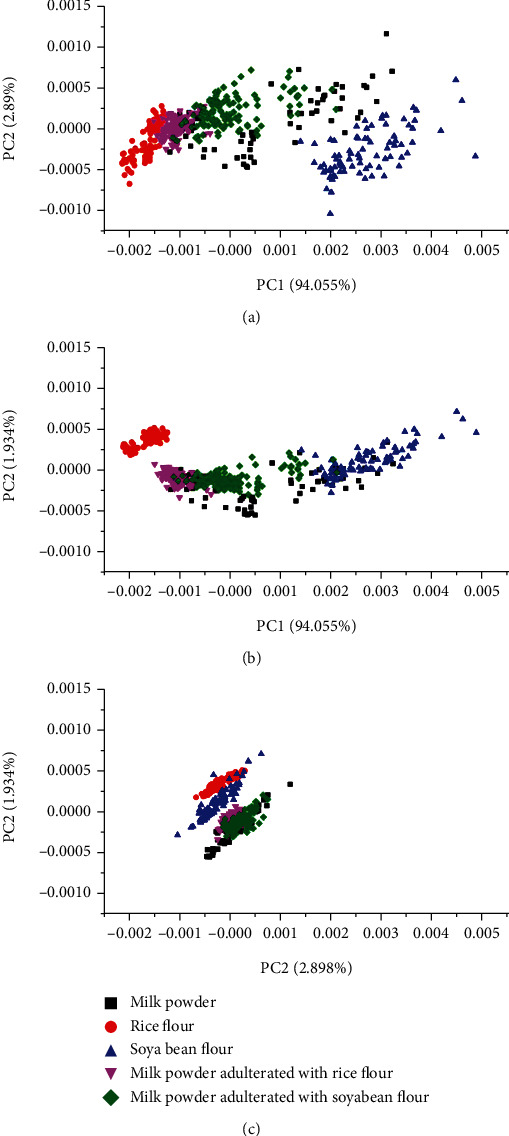
PCA score plot for classification of milk powder and adulterated milk powder with soybean flour and rice flour using PC1 vs. PC2 (a), PC1 vs. PC3 (b), and PC2 vs. PC3 (c) [23].

Combination of Fourier transform mid infrared spectroscopy and chemometrics has been employed for the detection of soybean and rice flour in milk powder. The presence of adulterants in milk powder was classified with PCA and quantified by using PLS, SVM (support vector machine), and ELM (extreme learning machine). The PCA model using the first three PC represented 98.888% of all variables. PCA could be used for classification of milk powder, milk powder adulterated with soybean flour, and milk powder adulterated with rice flour (Figure 1). Each sample was well separated and located in a specific cluster. Even though there are still overlaps among flour samples, in general, PCA could be used to identify the adulteration in milk powder. On the other hand, quantification of adulterants in milk powder was successfully performed using PLS, SVM, and ELM. All models demonstrated a high value of *R*^2^ either in calibration or in validation (>0.9) and low value of RMSEC and RMSEP (<3). This indicated that PLS, SVM, and ELM model demonstrated good accuracy and good precision for quantification of soybean flour and rice flour in milk powder [23].

FTIR spectroscopy using ATR technique has been used for the detection of soy milk in binary mixtures with other milks such as cow milk and buffalo milk. Milk samples, soy milk, and adulterated milks with soy milk were prepared and were dried using a freeze drying method. The FTIR spectra were measured in the wavenumber region of 4000-500 cm^−1^. PCA was successfully used for classification of cow milk, buffalo milk, soy milk, and adulterated milks with soy milk. All adulterated milks with soy milk were perfectly separated using PCA even for 5% adulterant concentration. The spectra were subjected to multiple linear regression (MLR) for the quantitative analysis of soy milk in cow and buffalo milks. A region of 1472-1241 cm^−1^ was the best range for predicting soy milk concentration. The MLR model demonstrated high *R*^2^ values in either the calibration (0.99) or validation (0.92) models [24].

Attenuated total reflectance-FTIR spectroscopy (ATR-FTIR) was investigated for adulteration analysis of pure ghee (PG) and heat clarified milk fat, with pig body fat (PBF). The combined wavenumber regions of 3030-2785, 1786-1680, and 1490-919 cm^−1^ were selected due to their capability for providing the highest level of classification between authentic PG and PG adulterated with PBF. PCA using absorbance values of these wavenumbers could classify both groups (authentic and adulterated) with the first (PC1) and second principle components (PC2) contributing to 82 and 18% variances, respectively. In addition, using SIMCA, approximately 90% of the samples could be classified according to its respective class. For quantification, PLSR could predict the levels of PBF in PG with *R*^2^ of >0.99 for the correlation model between actual and predicted values with a detection level of 1% [25].

## 3. Analysis of Foreign Ingredients in Milk

Various foreign components which can be safe such as allowed preservatives and sucrose or unsafe ingredients such as formalin and sucrose may be added into milk products. This act is considered adulteration practice, and therefore, some vibrational spectroscopic techniques have been used for the detection of this adulteration practice.

### 3.1. Analysis of Added Sucrose in Milk

Sucrose may be added into milk illegally in order to improve the total solid contents. FTIR spectroscopy combined with PLSR for quantification of sucrose in milk as well as PCA and SIMCA for classification of genuine cow milk and adulterated cow milk was used. FTIR spectra of all samples (pure milk and adulterated milk with sucrose at 0.5–7.5% *w*/*v*) were scanned in the spectral region of 4000–400 cm^−1^. The PLSR model using FTIR normal spectra at wavenumbers of 1070–980 cm^−1^ was used exhibiting the best prediction with *R*^2^ of 0.996 and 0.993, in the calibration and validation models, respectively. RMSEC and RMSEP values obtained were of 0.15% *w*/*v* and 0.20%, respectively. The limit of detection (LOD) value of sucrose was 0.5%. In addition, PCA has been successfully used for the discrimination of pure cow milk and adulterated cow milk, and SIMCA was capable of classifying both groups (authentic and adulterated) with a classification efficiency of 100% [26].

Raman spectroscopy in combination with PLS and PLS-DA was used for the analysis of sucrose added in full cream milk. All samples were measured using Raman spectrometer in a backscattered configuration with a 25 mW 785 nm laser. The obtained spectra were preprocessed prior to chemometrics analysis. Spectra were baseline corrected using 3-point baseline correction mode, and then, derivatization using second derivative Savitzky-Golay method was performed for smoothing. The LOD value obtained from Raman measurement was 7.205 mg/L whereas the LOQ (limit of quantitation) value was 21.834 mg/L. PLS has been successfully applied for sucrose quantification in full cream milk with minimum LOD threshold < 0.8%. The obtained *R*^2^ internal cross validation was 0.99, and the RMSECV value was 611. PLS-DA exhibited good classification ability to differentiate between pure full cream milk and full cream milk adulterated with sucrose. All samples containing sucrose were correctly classified as adulterated samples [27].

### 3.2. Analysis of Formalin

Formalin may be added into milk products illegally for increasing the shelf life of milk. The combination of raw FTIR spectra combined with chemometrics has been applied as rapid tools for the analysis of formalin in cow milk. Spectra of pure and adulterated milk (0.5–5% *v*/*v*) were scanned at 4000−400 cm^−1^ using accessory of attenuated total reflectance (ATR). Two multivariate calibrations of PLSR and PCR were compared using absorbance values at wavenumbers of 1080−950 cm^−1^, and the result showed that PLSR resulted in a better model. The PLSR model provided *R*^2^ of 0.977 for the correlation between actual values and calculated values in calibration models, with low RMSEC value (0.253% *v*/*v*) and low relative error (0.08). In addition, the validation model gave a *R*^2^ (prediction) value of 0.985, with RMSEP of 0.203% *v*/*v*, and relative percentage difference of 8.7. This method has LOD value of 0.5% of formalin. This indicated that the developed model had very good accuracy and precision. The chemometrics of PCA could differentiate pure samples from adulterated samples, while SIMCA could classify milk with and without formalin with accuracy efficiency of 100% [13].

The presence of formalin in cow milk was also analyzed using near-infrared (NIR) spectroscopy combined with chemometrics. Combination of NIR and chemometrics provided a good method for the detection and quantification of formalin in cow milk samples. The concentration of formalin used was in the range of 1-17%. The acquisition of NIR spectra was carried out in the wavelength of 2500-700 nm with resolution of 2 cm^−1^, and the spectra were recorded in absorbance mode. PLS-DA successfully differentiated between pure cow milk and cow milk added with formalin with high *R*^2^ value (0.969) and low RMSE value (0.086) which indicated good accuracy and precision of the model. The concentration of formalin in cow milk could be predicted perfectly using PLS regression resulting high *R*^2^ value (0.93%). PLS regression could detect and quantify formalin in the concentration level of less than 2%. Therefore, this method was sensitive enough for quantification analysis. Evaluation of PLS calibration using either internal or external validation confirmed the validity of the PLS regression model. Internal calibration using leave-one-out cross-validation technique resulted low RMSECV (1.38) whereas external calibration presented RMSEP of 1.50 indicating high validity [28].

Saha and Thangavel [29] applied Fourier transform near infrared (FT-NIR) in combination with multivariate analysis for the analysis of formalin in cow milk. The formalin concentration spiked in milk was in the range of 0-20%. Spectra acquisition was performed in the wavenumber of 12000-4000 cm^−1^ using resolution of 8 cm^−1^. The NIR spectra were subjected to preprocessing step prior to multivariate analysis in order to obtain a good chemometrics model. PLS calibration models were created in several wavenumber regions to obtain the optimum one. The PLS model built in the region of 6102-4246.7 cm^−1^ using 6 PLS factors and vector normalization processing method was chosen as the PLS calibration model for the quantification of formalin in cow milk samples because it demonstrated the highest *R*^2^ (0.9952) value and the lowest RMSEC (0.409) value among developed models. Moreover, a validation test using external validation also showed high *R*^2^ value (0.9954) and low value of RMSEP (0.427). It suggested that FT-NIR spectroscopy in combination with multivariate analysis has a strong potential to be used for the authentication of milk from formalin adulteration.

### 3.3. Analysis of Melamine

The protein contents in milk are used as metric quality and main parameter during milk production. The official and standard methods for protein contents in milk are Kjeldahl methods by determining nitrogen contents according to Association of Official Analytical Chemists' [30]. Addition of foreign compounds rich in nitrogen could increase the levels of proteins. Melamine has been reported to be illegally added into dairy products to increase the protein contents. FTIR spectroscopy combined with partial least square regression (PLSR) has been used for the quantification of melamine in milk (liquid and powder) using an accessory of single bound-attenuated total reflectance (SB-ATR). PLS models were established for developing the correlation between actual values of melamine and FTIR spectral absorbances at wavenumbers of 840–726 cm^−1^ with *R*^2^ of >0.99 as well as RMSEC and RMSEP of 0.370% and 1.51%, respectively. The method was linear at a calibration range of 0.0625-25% with LOD and LOQ of 0.00025% and 0.0015%, respectively. The proposed FTIR spectroscopy is rapid using an assay time of 1–2 min with little or without any sample preparation [31].

Near infrared (FT-NIR) and mid infrared (FT-MIR) were also used for the determination of melamine (2,4,6-triamino-1,3,5-triazine) in infant formula samples, powder samples, and liquid milk samples. Some spectral processing techniques such as mean centering, mean scattering correction, and spectral derivatization were tried and optimized to get the best prediction models. FT-NIR spectra were scanned at 1110–2500 nm corresponding to 9000 and 4500 cm^−1^, while FT-MIR spectra were scanned at 4000 and 500 cm^−1^. Using nonlinear methods, the correlation between actual values of melamine and FT-NIR/FT-MIR predicted methods revealed the valid results in which a LOD value of 0.76 ± 0.11 ppm could be reached using the correct preprocessing technique [27].

**Figure 2:**
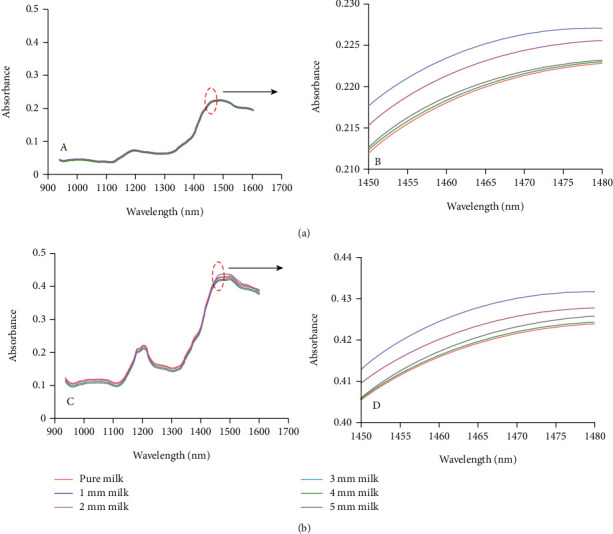
Raman spectra of milk powder and melamine measured using different penetration depth of milk samples prepared using “valley” nonfat milk (a) and “peak” whole milk (b). Plots A and C show the full spectra, while plots B and D show the enlarged spectra in the wavelength of 1466.3 nm [32].

The presence of melamine in milk powder has also been detected using near-infrared hyperspectral imaging by evaluating the penetration depth. Different thicknesses of milk powder (1-5 mm) were prepared and placed above the melamine layer for evaluating hyperspectral light penetration. Two types of milk powder were used, namely, nonfat milk and whole milk, and samples were measured at the wavelength of 937.5-1653.7 nm with 4.8 nm of average wavenumber spacing. The NIR spectra of pure milk powder and adulterated milk powder with melamine were very similar in a pattern (Figure 2). There is no specific peak of melamine observed; however, deep investigation at wavenumber of 1466.3 nm showed specific change as the depth of milk powder increased. The decrease in milk absorbance was obtained with the depth of milk powder from 1 to 3 mm whereas the spectra of melamine-milk powder and pure milk powder were very similar at the depth of 4-5 mm. It is presumed that hyperspectral NIR imaging could detect the presence of melamine using 1-3 mm sample depth [32].

NIR combined with chemometrics of pattern recognition including PLS-DA and SIMCA and chemometrics regression of PLS has also been employed for the analysis of melamine in milk powder. The pure and adulterated samples were subjected to a NIR spectrometer equipped with an optical fiber probe in the wavenumber of 10000-4000 cm^−1^ with 32 scan numbers. The variables used for chemometrics analysis were in the range of 5882-4000 cm^−1^. Melamine concentration is well predicted using PLS and uninformative variable elimination (UVE-PLS) models with high precision and accuracy. The obtained *R*^2^ value was 0.93 for PLS model and 0.97 for the UVE-PLS model. Internal validation using leave-one-out cross-validation technique displayed a low value of RMSECV which accounted for <0.5 in both the PLS and UVE-PLS models. Qualitative analysis using SIMCA could classify authentic and adulterated milk powder with melamine. However, misclassification occurred in lower adulterant concentration. Investigation using the PLS-DA model showed better classification compared to SIMCA. PLS-DA correctly classified all adulterated samples even in lower melamine concentrations. Therefore, PLS-DA is highly recommended for the classification of melamine adulteration in milk products [33].

Enhanced Raman Scattering spectroscopy (ERSS) was used for the determination of melamine in milk matrices using selective binding of melamine with gold nanoparticles (AuNPs) which promote the aggregation of AuNPs inducing a huge enhancement of melamine signals in Raman spectrum. The highest intensity of melamine in Raman spectra was observed at peak of 715 cm^−1^; therefore, this peak was selected for the prediction of melamine. The method was linear at a concentration range of 0.31–5.0 mg/L with a *R*^2^ value of 0.99. The values of LOD and LOQ were 0.017 and 0.057 mg/L, respectively, in the milk extracts which correspond to values of 0.17 mg/L (LOD) and 0.57 mg/L (LOQ) in the milk matrix. The accuracy of method as described by mean recovery resulted in percentage recovery of 99.9% and 96.3% in samples spiked by low and high levels of melamine, respectively. In addition, the RSD values for precision study were of 9.6% and 3.8% samples spiked with low and high levels of melamine, respectively. This method is simple and rapid and does not require a long extraction procedure for the detection of melamine in milk samples [34].

### 3.4. Analysis of Urea in Milk

Urea is often added into milk products in order to obtain more concentrated milks by increasing the solid nonfat (SNF) value. The presence of urea in milk is normal; however, the acceptance limit of urea concentration is approximately 70 mg/dL [35, 36]. High consumption of urea is related to several health problems. Therefore, it is very important to ensure the quality of milks by analyzing the amount of urea presented in milks. A high accuracy and precision analytical method instead of lactometer is highly required to detect and quantify the presence of urea in milks because a lactometer often failed to differentiate between pure milk and milk added with urea. A lactometer tests the purity of milk by measuring relative density of milk with respect to water. Urea is one of compounds which can be used to increase relative density of milk; therefore, it increases the lactometer reading [37]. A lactometer recognizes this condition as a good purity milk; as a consequence, other methods are required to detect the presence of urea in milk. Raman spectroscopy was successfully used for the analysis of urea in milk. Combined with PLS, it can be used for the quantification of urea in milk. The concentration of urea added into the milk was in the range of 10-1000 mg/dL. The acquisition of Raman spectra was carried out with Raman spectroscopy equipped with a 785 nm diode laser. Data preprocessing steps including spectra correction, derivatization using Savitzky-Golay method, and binning were carried out prior to PLS analysis to obtain good variation of variables. The PLS model using wavenumber range of 1800-750 cm^−1^ was successfully applied for quantification of urea in milk. The model showed high *R*^2^ (>0.99) in both the calibration and validation models. Obtained RMSEC was 39.71 ± 5.50 mg/dL while the RMSEP was 43.89 ± 6.01 mg/dL. The developed PLS model indicated high accuracy because it can detect the urea samples in milk with accuracy more than 90% [38]

The presence of urea in cow and buffalo milk (ratio of 1 : 1) was also detected using FTIR-ATR spectroscopy combined with pattern recognition and multivariate calibration. The urea added into milk was in the concentration range of 100-2000 ppm. The spectra were recorded in the wavenumber region of 4000-700 cm^−1^ using resolution of 4 cm^−1^ and 24 scan numbers. The FTIR spectra of authentic and adulterated milk with urea could be distinguished because they exhibited different spectral pattern especially in the region of 3600-2800 cm^−1^ and 1670-1564 cm^−1^. The peaks presented in the 1670-1564 cm^−1^ associated with the peaks of the CO, CN, and NH_2_ groups in urea [39, 40]. PCA could differentiate between authentic and adulterated milks with urea. There were three separated groups observed in the PCA score plot, namely, authentic cow-buffalo milk, adulterated milk with urea (concentration of 100-900 ppm), and adulterated milk with urea (concentration of 1300-2000 ppm). Another chemometrics classification method, namely, SIMCA (soft independent modeling class analogy), perfectly classified authentic cow-buffalo milk and adulterated milks with urea. All adulterated samples well separated from authentic cow-buffalo milk. Quantification of urea in cow-buffalo performed using the PLS calibration model resulted maximum *R*^2^ for calibration and validation of 0.9 and 0.86, respectively, whereas the RMSEC value was 183.77 ppm and the RMSEP value was 254.23 ppm. It was suggested that FTIR spectroscopy combined with chemometrics was adequate enough for analysis of urea in milk samples [14]

### 3.5. Determination of Geographical Origin

Authentication of bovine milk was evaluated by determining the origin of fresh grass feeding, pasture grazing, and organic farming using FTIR spectroscopy and chemometrics. The samples were obtained by taking bovine milks from the cows on pasture, with the presence or absence of fresh grass in different farming systems of organic and biodynamic or conventional type. Previously, the samples were subjected to PCA at MIR spectral regions of 900–3000 cm^−1^ with the exclusion of regions of 1800–2800 cm^−1^, intended to reveal natural clustering of the samples and to detect outliers. Some PLS-DA regression vectors exhibited high scores for bands at around 1/*λ* 1640 cm^−1^, 1585 cm^−1^, and 1695 cm^−1^, smaller peaks around 1020 cm^−1^ and 1380 cm^−1^ and at 1/*λ* 3000–2800 cm^−1^. Bovine milk classification for the prediction of fresh grass feeding, pasture grazing, and organic farming was carried out using partial least square discriminant analysis (PLS-DA) using variables of absorbance values of FTIR spectra at certain wavenumbers. The PLS-DA model could discriminate bovine milk from cows feeding fresh grass and not fresh grass with sensitivity and specificity values of 88% and 83%, respectively. PLS-DA was also able to discriminate bovine milk pasture grazing (indoors versus outdoors). Discrimination of organic and conventional bovine milk could be accomplished with PLS-DA providing acceptable accuracy of 80% and 94% in training and validation sets, respectively. From this result, FTIR spectra could contain valuable information on bovine milks from on cows differing in diet which could be used for authentication purposes [15].

## 4. Conclusion

Detection and analysis of milk adulteration are very challenging due to the wide ranges of adulterant types. The development of rapid and high reproducible method is highly important for milk authentication. Vibrational spectroscopy including Fourier transform infrared (FTIR), near-infrared (NIR), and Raman spectroscopy revealed promising analytical techniques for screening and identification of other substances in milk products. Optimization of wavenumber region of FTIR, NIR, and Raman could be used for the analysis of specific adulterants in milk samples. Combined with chemometrics techniques including pattern recognition and multivariate calibration, vibrational spectroscopy methods emerge as promising, rapid, and reliable analytical tools for the authentication of milk products with high precision and high accuracy.

## Figures and Tables

**Table 1:** The application of vibrational spectroscopy (Raman, near infrared, and mid infrared) combined with chemometrics for milk authentication.

	Adulteration issues	Type of vibrational spectroscopy	Chemometrics	Results	Ref.
Cow milk	Addition of sucrose to cow milk	Normal mid IR spectra at wavenumbers of 1070–980 cm^−1^	PCA and SIMCA for classification. PCR and PLS for quantification	The levels of sucrose cold be quantified with *R*^2^ Cal: 0.996; *R*^2^ Val: 0.993, RMSE (Cal: 0.15% *w*/*v*; Val: 0.20% *w*/*v*), RE% (Cal: 4.9% *w*/*v*; Val: 5.1% *w*/*v*), and RPD (13.40). SIMCA was able to classify test samples with a classification efficiency of 100%	[4]
Raw milk	Detection of reconstituent milk powder in milk	First derivative spectra at wavenumbers of 800-1800 cm^_1^	PCA and PLS-DA for classification	FTIR spectroscopy has great potentials in quality control of milk and their related products because the PLS-DA model yielded satisfactory separation of the two spectral fingerprints	[12]
Goat milk	Adulteration of goat milk with cow milk	MIR: 1373, 1454, and 956 cm^−1^ Raman: 1005, 1154, and 1551 cm^−1^	SIMCA for classification and PLSR for prediction of milk adulteration	SIMCA result showed the *β*-carotene band at 1373, 1454, and 956 cm^−1^ (MIR spectra) and 1005, 1154, and 1551 cm^−1^ (Raman spectra) as a biomarker for classification of cow milk in goat milk PLSR using MIR and Raman spectra were used to predict goat and cow milk mixtures with 0.32 SECV, 0.98 *R*^2^ Cal, 0.57 SEP, and 0.98 *R*^2^ Val (MIR) and 0.46 SECV, 0.96 *R*^2^ Cal, 0.57 SEP, and 0.94 *R*^2^ Val (Raman)	[21]
Mengniu milk, Yili milk, and Haihe milk	Addition of melamine in milk	2D IR/NIR heterospectra range of 1400-1704 cm^−1^ and 4200-4800 cm^−1^	NPLS-DA for classification of pure milk and adulterated milk	Results showed that, for the samples in the prediction set, the rate of correct classification was 96.2% using synchronous 2D heterospectra IR/NIR correlation spectra versus 88.5% using synchronous 2D homospectral IR/IR and NIR/NIR correlation spectra. Comparison of the results showed that 2D heterospectra IR/NIR correlation spectra and NPLS-DA could give better classification between adulterated milk and pure milk	[41]
Raw cow milk	Addition of five common adulterants (water, starch, sodium citrate, formaldehyde and sucrose) in raw cow milk	MID infrared-ATR spectra range of 4000-600 cm^−1^	PLS-DA	The method was able to detect the presence of the adulterants water, starch, sodium citrate, formaldehyde, and sucrose in milk samples containing from one up to five of these analytes, in the range of 0.5–10% *w*/*v*	[42]
Raw cow milk	Addition of pseudo protein (urea, melamine, and ammonium nitrate) and thickeners (dextrin and starch)	First derivative NIR spectra at wavenumbers of 4000-10.000 cm^−1^	Nonlinear supervised pattern recognition methods of improved support vector machine (I-SVM) and improved and simplified K nearest neighbours (IS-KNN)	Both methods (I-SVM and IS-KNN) exhibit good adaptability in discriminating adulterated milks from raw cow milks at the concentration of adulteration solutions which equals or exceeds 5%	[25]
Nescafe milk powder	Addition of melamine	Normal NIR spectra at wavenumbers 4000-10.000 cm^−1^	One class partial least square (OCPLS)	The combination of NIR spectroscopy and OCPLS can serve as a potential tool for rapid and on-site screening melamine in milk samples with the total accuracy of 89%, the sensitivity of 90%, and the specificity of 88%	[26]
Infant formula (powder), milk powder, and milk liquid	Addition of melamine	NIR spectra range of 9000-4500 cm^−1^ MIR spectra range of 500-4000 cm^−1^	Partial least square (PLS), orthogonal projection to latent structures (OPLS), polynomial partial least squares (Poly-PLS), artificial neural networks (ANN), and support vector machine (SVM)	Linear calibration methods (PLS and OPLS) show a much larger prediction error, exceeding 1 ppm. The average error of the PLS/OPLS methods is 31 ± 0.07 ppm, while the error of the Poly-PLS, ANN, and SVM-based methods is almost 5 times smaller (0.28 ± 0.05) The relationship between the MIR/NIR spectrum of milk product and melamine content is nonlinear. Thus, nonlinear regression methods, such as Poly-PLS, ANN, SVR, or LS-SVM, are needed to correctly predict the melamine	[27]
Cow milk	Milk adulterated with formaldehyde, hydrogen peroxide bicarbonate, carbonate, chloride, citrate, hydroxide, hypochlorite, starch, sucrose, and water	MIR region at wavenumbers of 1000-4000 cm^−1^	Multiplicative scatter correction (MSC) for spectra preprocessing; PCA for visualization of the sample distribution, SIMCA for classification milk	In the first step, a one-class model was developed with unadulterated samples, providing 93.1% sensitivity. Four poorly assigned adulterants were discarded for the following step (multiclass modelling). Then, in the second step, a multiclass model, which considered unadulterated and formaldehyde, hydrogen peroxide, citrate, hydroxide, and starch as adulterated samples, was implemented, providing 82% correct classifications, 17% inconclusive classifications, and 1% misclassifications	[43]
Cow milk	Tetracycline's residue (tetracycline, chlortetracycline, and oxytetracycline)	MID FTIR spectra at wavenumber of 4000-550 cm^−1^	SIMCA for classification, PLS and PCR for quantification of tetracycline residue	SIMCA could be used for classification of pure milk and milk adulterated with the confidence level of 99%. The calibration models developed with three algorithms (PLS1, PLS2 and PCR) to predict tetracycline, chlortetracycline, and oxytetracycline concentrations in milk revealed values of *R*^2^ of 0.999, 0.998, and 0.997, respectively	[44]
Raw milk	Addition of tetracycline	FT-MIR spectra at wavenumber of 1550-1725 and 2800-2981 cm^−1^, while FT-NIR used raw and first derivative spectra at the region of 3500-8000 cm^−1^	PLS for quantification of tetracycline hydrochloride in milk	FT-MIR: the optimum number of factors using PLS method was 15, and the *R*^2^ between the predicted and actual values was 0.89, the SEC value was 385 ppb, and the repeatability value was 163 FT-NIR: PLS-first derivative calibration method gave an *R*^2^ value of 0.76, SEC value of 431 ppb, and the repeatability value of 73 ppb Results indicated that FT-MIR spectroscopy could be used for rapid detection of tetracycline hydrochloride residues in milk	[45]
Pasteurized milk	Addition of sweet whey in milk	Raman spectra in range from 800-1800 cm^−1^	ANN for quantification and PLS for corrected prediction	A high-capacity prediction model was obtained using ANN, with *R*^2^ of 0.9999. Alternatively, ANN can be replaced by a linear model adjusted using PLS, which also exhibited reasonable results for the prediction of percentage of whey added (*R*^2^ = 0.99)	[46]
Cow milk	Addition of water, urea, starch, and goat milk	NIR spectra in region of 950-1650 nm	PCA and the data driven soft independent modeling of class analogy (DD-SIMCA) for classification, PLS for quantification	Preliminary PCA performed on the whole data revealed that both big similarities and differences between pure and adulterated milk samples were collected from a variety of dairy farms The DD-SIMCA approach achieved satisfactory classification. By the PLSR model, standard error of prediction (SEP) values of 4.35, 0.34, 4.74, and 5.56 g/L and *R*^2^ Val value of 0.94, 0.87, 0.93, and 0.89 were obtained for water, urea, starch, and goat milk, respectively	[47]
Cow milk	Real time prediction of fat, protein, and lactose	NIR spectra in region 950-1690 nm	PLSR for quantification of fat, protein, and lactose	The obtained prediction models were thoroughly tested on all the remaining samples not included in the calibration sets (*n*, respectively, 846 and 857). For the post hoc prediction models, this resulted in an overall prediction error (RMSEP) smaller than 0.08% (all % are in *w*/*w*) for milk fat (range 1.5-6.3%), protein (2.6-4.3%), and lactose (4-5.1%), while for the real-time prediction models, the RMSEP was smaller than 0.09% for milk fat and lactose and smaller than 0.11% for protein	[48]
Cow milk	Adulteration with water or whey	Second derivative NIR spectra (whole region, 1100-1850, 2048-2500, and combination of 1100-1850, 2048-2500 nm)	DPLS and SIMCA for classification, PLSR for quantification	The best DPLS classification model for natural milk, milk adulterated with water and milk adulterated with whey was developed using the MSC and second derivative spectra in the whole region of 1100–2500 nm with a PLS factor of 7 and classification performance of 100% The best prediction result is obtained for water adulterated in natural milk, when the model is developed by using the MSC spectra over the whole region of 1100–2500 nm. Its statistical results are the lowest value of the root mean square error of prediction (RMSEP) of 2.159% (*v*/*v*) with a PLS factor of 4, while the best calibration model for milk adulteration by mixing whey yields the prediction result with a RMSEP value of 0.244% (*g*/*v*) by a PLS factor of 4. This model was built using the MSC pretreated spectra of the combination regions of 1100–1850 and 2048–2500 nm	[49]
Commercial milk samples	Adulteration with water	NIR spectra at 400-2500 nm	PCA for classification and PLS for quantification	PCA perfectly classified between pure milk and milk adulterated with water. PLS was successfully used to predict the concentration of water in milk samples with *R*^2^ more than 0.9 and RMSEC lower than 0.04	[50]
Cow milk	Hydrogen peroxide	FTIR spectra at 4000-600 cm^−1^	Artificial neural network (ANN) for classification and multiple linear regression (MLR) for quantification	Chemometrics of ANN could classify pure and adulterated milk samples with hydrogen peroxide with high accuracy. Quantification of hydrogen peroxide could be obtained using MLR with *R*^2^ of calibration 0.80 and RMSEC value of 0.15	[51]
Raw milk	Sodium hypochlorite	FTIR spectra at 4000-650 cm^−1^	SIMCA for classification	SIMCA could classify pure raw milk and adulterated raw milk with sodium hypochlorite with a specificity of 56.7%	[43]

## Abbreviations

2D: Two-dimensional
ANN: Artificial neural networks
ATR: Attenuated total reflectance
DD-SIMCA: Data driven soft independent modeling of class analogy
DPLS: Discriminant partial least squares
ERSS: Enhanced Raman Scattering spectroscopy
FTIR: Fourier transform infrared
I-SVM: Improved support vector machine
IS-KNN: Simplified K nearest neighbours
iSPA-PLS: Interval selection in PLS
LOD: Limit of detection
LOQ: Limit of quantification
MDA: Multivariate data analysis
MID IR: Mid infrared
MIR: Mid infrared
MLR: Multilinear regression
MSC: Multiplicative scatter correction
NPLS-DA: Multiway partial least squares discriminant analysis
OCPLS: One class partial least squares
OPLS: Orthogonal projection to latent structures
PCA: Principal component analysis
Poly-PLS: Polynomial partial least squares
PLS-DA: Partial least square-discriminant analysis
PLSR: Partial least square regression
RMSE: Root meat square error
RMSEP: Root mean square error of prediction
RMSEC: Root mean square error of calibration
*R*^2^ Cal: Coefficient of determination of calibration
*R*^2^ Val: Coefficient of determination of validation
SB-ATR: Single bound-attenuated total reflectance
SIMCA: Soft independent modeling of class analogy
SEC: Standard error of calibration
SECV: Standard error of calibration
SEP: Standard error of prediction
SNF: Solid nonfat
SVM: Support vector machine
UVE-PLS: Uninformative variable elimination partial least square.
